# Decoding the mechanism of hypertension through multiomics profiling

**DOI:** 10.1038/s41371-022-00769-8

**Published:** 2022-11-03

**Authors:** Eric Adua

**Affiliations:** 1 School of Clinical Medicine, Medicine & Health, Rural Clinical Campus, University of New South Wales, Wagga Wagga, NSW Australia; 2 School of Medical and Health Sciences, Edith Cowan University, Joondalup, WA Australia

**Keywords:** Hypertension, Risk factors, Medical research

## Abstract

Hypertension, characterised by a constant high blood pressure, is the primary risk factor for multiple cardiovascular events and a major cause of death in adults. Excitingly, innovations in high-throughput technologies have enabled the global exploration of the whole genome (genomics), revealing dysregulated genes that are linked to hypertension. Moreover, post-genomic biomarkers, from the emerging fields of transcriptomics, proteomics, glycomics and lipidomics, have provided new insights into the molecular underpinnings of hypertension. In this paper, we review the pathophysiology of hypertension, and highlight the multi-omics approaches for hypertension prediction and diagnosis.

## Introduction

Underlying the significant improvement of living conditions coupled with the ever-changing global society is the increasing prevalence of cardiovascular events. This is especially true for hypertension, an asymptomatic condition marked by increased blood volume, sustained vasoconstriction, and vascular resistance, which ultimately raises blood pressure. The current estimate of hypertension prevalence is about 1.13 billion worldwide, and an exponential increase in the personal, social, and financial burden is expected as the population ages globally [1]. Besides causing the death of an approximately 8.5 million people with stroke, ischemic heart disease, vascular and renal disease, hypertension also fuels other pathological conditions including atherogenesis and multi-infarct dementia [2]. In addition, it accelerates the degeneration of vessel walls that result in aortic dissection and vascular haemorrhage [3].

High blood pressure results from the interplay between genetic and environmental factors. From the perspective of genetic factors, hypertension has been associated with single gene mutations that code for proteins involved in sodium and water reabsorption [3]. Indeed, genetic factors are responsible for 30–60% of an individual’s risk [4]. Hypertension, like other cardiovascular diseases, is a familial disease. This is exemplified in the Framingham Heart Study where blood pressure data on three generations were studied. It was shown that children whose parents and grandparents had hypertension or raised blood pressure had increased risk of developing the condition even in the absence of multiple environmental factors [5, 6]. Further, a study revealed an increased correlation between monozygotic twins and blood pressure, when compared to dizygotic twins [7]. On the other hand, environmental factors that increase the risk of hypertension include stress, increased salt intake, physical inactivity, and obesity [3]. Moreover, hypertension is the consequence of the interaction of vascular cells such as endothelial cells and arterial smooth muscle cells [3]. The association between age and hypertension has also been reported, with ageing linked to vascular injury and endothelial dysfunction [8]. The multifactorial and asymptomatic nature of hypertension complicates its detection, and prediction of those who are likely to develop the condition is uncertain. Shockingly, 580 million people are unaware of their status and hence are not receiving treatment. The American Heart Foundation has set the following criteria for the diagnoses of hypertension. Stage 1 hypertension is systolic between 130–139 or diastolic between 80–89; Stage 2 is systolic of at least 140 or diastolic at least 90 mm Hg [9]. Thus, persistent elevation beyond these thresholds is indicative of hypertension. The reasons for poor blood pressure control include medication side effects, poor patient provider relationship, non-adherence to medications, obesity, family history and the presence of comorbidity [10, 11]. Alongside changes in lifestyle, effective antihypertensive medicines, either administered as a monotherapy or dual therapy/single-pill combination medicine, can potentially restore blood pressure to normal thresholds. Yet, SBP/DBP is below 140/90 mmHg only in less than 14% adults worldwide, and less than 8% in developing countries [12]. Undoubtedly, early detection will drive better outcomes, while forestalling hypertension development and its associated complications. A study has shown that quality health education, good knowledge and practices relating to hypertension can improve BP control [13]. Health professionals need to be trained on hypertension management to ensure effective communication and proper dissemination of information. There must be a paradigm shift from the consumption of unhealthy diets such as high salt foods, processed and high-fat diets to more healthy choices such as fruits and vegetables. Taken together, these practices also underpin the concept of predictive, preventive, and personalised medicine (PPPM).

PPPM is a new concept that integrates patient-specific information and large amounts of data including clinical, registered-based, and monitoring data to predict an individual’s predisposition toward a disease, propose preventive measures and develop treatments that meet the individual’s health care needs [14, 15]. PPPM optimises and complements current healthcare settings by exploring biomarkers in human biological samples including plasma, serum, and urine. Genetic or genomic analysis, has been central to PPPM, allowing exploration of global gene expression patterns [16]. The integrative genetics has thus far, also paved the way for emerging fields including transcriptomics, proteomics, lipidomics and glycomics [17, 18]. Enabled by high-throughput technologies and robust statistical tools, these fields have identified potential biomarkers for hypertension and provided useful information about the aetiology and progression of the disease [19–21]. In the current study, we review the pathophysiology of hypertension and explore multi-omics profiling for the detection and prediction of hypertension. Each of these technologies has its strengths and weaknesses. As such, this review provides technological advances in multi-omics profiling.

## Pathophysiology of hypertension

Hypertension is a complex condition and understanding its pathophysiology has implications for treatment and delay of complications. Baroreflex through the action of stretch receptors on carotid sinus and aorta, provides a negative feedback loop that regulate blood pressure. When blood vessels stretch due to high blood pressure, baroreceptors are activated which in turn send signals to the nucleus tractus solitarius (NTS) in the brain stem via the glossopharyngeal nerve and vagus nerve. Consequently, the parasympathetic nervous system (PNS) is activated, releasing acetylcholine that acts on the pacemaker cells in the sinoatrial node. This effectively results in vasodilatation, reduction of heart rate and normalisation of blood pressure [22]. On the other hand, baroreceptors detect a decrease in blood pressure and sends signal to the NTS, which acts by deactivating PNS and activating the sympathetic nervous system (SNS). Through the action of catecholamines, efferent fibres of SNS increase heart rate, cardiac output and constrict blood vessels, alongside an increase in resistance, decrease in blood flow, and ultimately, an increase arterial blood pressure. Efferent fibres of the SNS also activate the kidney to secret renin that triggers the renin angiotensin aldosterone system (RAAS).

Under physiological conditions, the RAAS is activated in response to a decrease in renal blood pressure, and effectively restores systemic blood volume and systemic blood pressure [23]. Specifically, the juxtaglomerular cells release renin into the blood. Once in the blood, renin acts on angiotensinogen from the liver, converting it to its active form, angiotensin. Catalysed by angiotensin converting enzyme from the lungs, angiotensin I is converted to angiotensin II. Angiotensin II binds to angiotensin II type I & 2 receptors in the adrenal cortex, kidney, and arterioles. When bound, angiotensin II elicits multiple effector functions including vasoconstriction and increase Na^2+^ reabsorption in the kidneys by aldosterone [3, 24, 25].

**Fig. 1:**
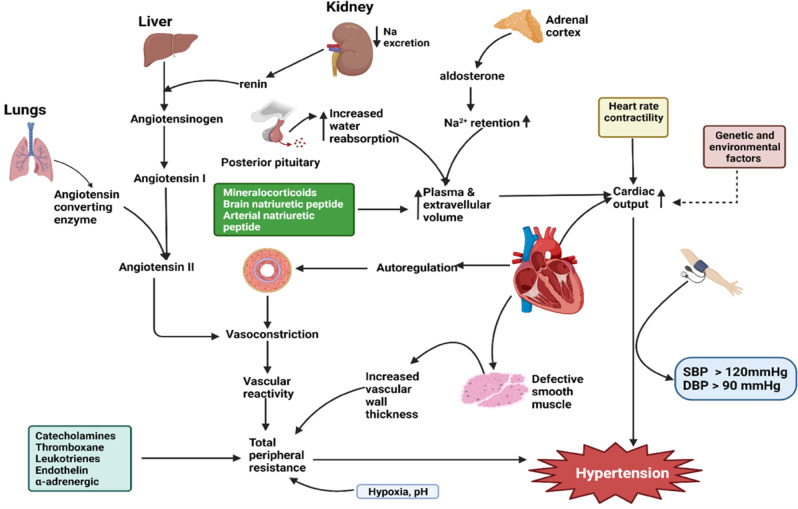
The role of multiple factors in the regulation of blood pressure. Blood pressure is the product of the cardiac output and peripheral vascular resistance. Peripheral resistance can be influenced by local factors such as pH, hypoxia, and humoral factors (angiotensin II, catecholamines, thromboxanes etc) whereas cardiac output is influenced by blood volume, atrial and brain natriuretic peptides and cardiac factors (heart rate and contractility). In response to decreased systemic blood pressure, renin is produced from the juxtaglomerular cells of the kidney, which stimulates the activation of angiotensinogen in the liver to angiotensin I. Angiotensin I is converted to angiotensin II by angiotensin converting enzyme from the lungs. Angiotensin II constricts blood vessels and promotes the release of aldosterone from the adrenal glands, which subsequently triggers sodium reabsorption. Antidiuretic hormones from the pituitary glands are also released to cause water reabsorption. Combined, these leads to increased blood volume and blood pressure.

However, in arterial hypertension, baroreflex mechanism is adjusted to a higher set point that sustains hypertension rather than suppressing it. Moreover, genetic, and environmental factors, reduced vessel wall extensibility and uncoupling of receptors to vessel wall may result in decreased baroreceptor sensitivity. Parasympathetic tone is reduced whereas SNS is overstimulated [22, 26]. In addition, overproduction of angiotensin II, impaired activities of vasodilators such as nitric oxides, natriuretic peptides, and prostacyclin **(**Fig. 1**)** may result in hypertension.

Both RAAS and SNS can influence the function of vascular smooth muscle cells, leading to vasoconstriction and hypertension. Vascular smooth muscle contraction depends on intracellular calcium levels and begins when a neurotransmitter such as acetylcholine and a vasoactive agent like angiotensin II binds and activates receptors of phospholipase C (PLC), yielding inositol triphosphate (IP_3_) and diacylglycerol (DAG). IP_3_ and DAG trigger the release and mobilisation of Ca^2+^ from the sarcoplasmic reticulum and promote cell proliferation, whereas DAG stimulates protein kinase C (PKC) [27]. With other Ca^2+^ channels opened, Ca^2+^enters the cells and accumulate. Ca^2+^ then binds to calmodulin, forming a calcium-calmodulin complex [28, 29]. This complex activates myosin light chain (MLC) kinase, stimulating cross bridge formation (interaction between myosin head and actin), thereby leading to shortening of myofibrils and contraction. Vascular smooth muscles are also stimulated by oxidative stress, haemodynamic changes and mechanical forces [30]. Other characteristics of hypertension are endothelial dysfunction and increased arterial stiffness [28, 29].

## Genetics and genomics

Genomics is a branch of medicine that studies the structure, function, editing and alterations in the genome whereas genetics studies individual genes. Genes have been identified to underlie key mechanisms in the pathophysiology of hypertension including those involved in the RAAS, catecholamine/adrenergic system, renal kallikrein–kinin system, epithelial sodium channel, adducin, and those involving lipoprotein metabolism, hormone receptors, and growth factors. Specifically, M235T allele of the angiotensinogen gene was linked to an increased risk of hypertension in two separate studies involving 27, 906 individuals [31, 32]. Among Europeans and Japanese, AGT variant (C4072T) was associated with hypertension. Gly16 mutation 2-adrenergic receptor gene was shown to cause a decrease in catecholamine vasodilatory responses in humans, suggesting the potential role of the 2-adrenergic receptor gene in the control of peripheral blood flow and arterial pressure. Chromosome 5q31-q34, locus for adrenergic receptors including 1B (ADRA1B), 2 (ADRB2), and dopamine D1 receptors, have been implicated in in blood pressure regulation (DRD1) [33]. In a case control study, A44221G of AGTR1 was shown to be associated with hypertension in African Americans in a single-locus analysis with the G allele increasing hypertension risk. Moreover, when African Americans and European’s were combined, the study found a significant association between *AGTR1* A44221G, *REN* C-4021T, *ACE* C8342T, A12292G, and A15990G and hypertension [34]. Monogenic hypertension has been the focus of genetic studies for years. This form of hypertension is due to single germline mutations that affect the functions of mineralocorticoid, glucocorticoid and androgens released from the adrenal cortex. For example, glucocorticoid-remediable aldosteronism (GRA) is an autosomal disorder that occur when the promoter region of 11 β-hydroxylase gene (CYP11B1) and the coding regions of the aldosterone synthase (CYP11B2) gene unequally crosses over on chromosome 8q. Hypertension can also be due to a mutation of epithelial sodium channel (ENaC), that cause Na^+^ reabsorption independent of aldosterone. Located on the distal nephron, ENaC is tightly regulated by aldosterone and antidiuretic hormone (ADH). Along with Na^+^/K^+^ ATPase, the ENaC ensures the homeostatic regulation of electrolytes. This channel has three subunits, encoded by SCNN1A, SCNN1B and SCNN1G, respectively. Germline mutations in these genes result in an increased channel opening probability, leading to increased Na^+^ reabsorption, volume expansion and hypertension. Although studies of monogenic forms of hypertension have provided insights into the aetiology of the condition, it is now clear that hypertension is polygenic nature.

Genome-wide association studies (GWAS) have transformed the study of complex disease genetics by testing millions of genetic variants throughout the genomes of individuals to find genotype–phenotype relationships [35]. Powered by GWAS, multiple single nucleotide polymorphisms (SNPs)—polymorphisms in the coding and promoter regions of the genome, can be identified. SNPs can affect gene expression and the function of proteins. Whereas SNPs may not cause a change in the phenotype, a nonsense variant may result in a pathological condition [36, 37]. In the context of hypertension, SNPs could affect blood pressure regulation in multiple aspects including effects on vascular endothelial function, cardiac function, and ion transport in the kidney [38]. In 2005, the first GWAS for age-related macular degeneration (AMD) was published. Following this, over 50,000 genome-wide significant correlations between genetic variations and prevalent diseases have been reported [39].

Newton-Cheh [40] published a large-scale GWAS results of 34,433 individuals of European ancestry from the Global BPgen consortium. The study tested 2.5 million genotyped and imputed SNPs for associations with systolic and diastolic blood pressure. They found that DBP and SBP were associated with CYP17A1, CYP1A2, FGF5, SH2B3, MTHFR, c10orf107, ZNF652, and PLCD3 genes (*P* = 7 1024, *P* = 1 1021, *P* = 3 1018, *P* = 2 1013, *P* = 5 109, *P* = 1). With 200,000 people of European origin in 2011, the International Consortium for Blood Pressure (ICBP) replicated the 13 prior loci successfully and found 16 novel loci that were significant at the genome-wide level [41]. By 2017, Warren et al. [42], had conducted a GWAS on 330,956 and identified 107 significant loci. Of the identified loci, 24 were linked to SBP, 41 to DBP, and 42 to pulse pressure (PP). In another large-scale meta-analysis study that considered smoking behaviour discovered loci associated with DBP and SBP in 610, 091 individuals. Up to 18.8 million SNPs were analysed in stage 1, along with the analysis of insertion/deletions in 129, 913 individuals from Hispanic, European, African and Asian Origin. The study found 15 loci to be significant in stage 1, which was confirmed in the stage 2 analysis. This significant loci increased to 66 in the combined stage 1 and 2 analysis [42]. GWAS analysis on 130,777 Asians individuals and subsequent meta-analysis on 289, 038 revealed 13, 000 SNPs to be associated with blood pressure phenotypes including SBP, DBP, mean arterial hypertension and PP. In addition, 19 new loci, 15 of which were found to be linked to Asian population [43].

Variations in both coding and non-coding regions of the genome may range from minor changes to large chromosomal defects. Either way, the function of the gene may be impacted. The variants that contribute to population variation of quantitative traits such as BP remains unclear. Even though BP has a high heritability, we still do not know whether common variants with modest effects or rare variants with big effects are responsible for the allelic variation in hypertension. It has been reported that common genetic variants explain only a fraction of heritable trait variation [44, 45]. For accurate estimation of SNP heritability, assumptions of how heritability is spread across the genome must be made. Undiscovered common variants (relatively high minor allele frequency), rare variants (relatively low minor allele frequency), non-addictive genetic variation, and epigenetic factors are only contributors of missing heritability. Thus, it is important to quantify common SNPs that contribute to variations [38]. In contrast to common variants, rare variants (<1%) have distinctive features such as lower linkage disequilibrium, and larger population specificity. Rare variants analysis often restricted to the coding regions of the genome from exome sequencing analysis and exon studies chip studies have discovered loci that are associated with BP. In a large-Scale WGS study that sought to determine signals of SBP, DBP, and hypertension among a multiancestry sample of 51,456 participants from the TOPMed and Center for Common Disease Genomics programs, Kelly et al., 2022 found that rs1462610506 at the novel LOC100506274, was associated with reduced SBP in stage-1 analysis but not in stage-2 analysis (*P* = 0.11). Moreover, in stage 1, rs36136513 in the INSR locus, was associated with DBP (beta [SE] = − 0.36 [0.07]; *P* = 4.18 × 10^−7^) [46].

GWAS have identified genetic variants in hypertension and have enabled an understanding of the traits that underlie hypertension. Sixty-four validated BP loci were reported in 2015 which rose dramatically to 1,477 few years later. In a study that analysed over 1 million people of European ancestry, Evangelou et al. [47], found more than 1000 independent signals for BP traits at 901 loci, including the discovery of 535 new loci. Despite the discovery of huge BP loci, the genetic variance explained by all loci combined is still low. The SNPs can explain up to 7% of variations in SBP and 27% of the 30–50% heritability of hypertension phenotype [47]. In two population-based European cohorts, Org et al. [48] discovered a single variant at the CDH13 (cadherin 13 preprotein) locus (rs11646213) that correlated with blood pressure and hypertension, while trying to replicate the 80 strongest associations with blood pressure and hypertension (hypertension, *p* = 5.30 × 10^−8^, SBP, SBP, *p* = 5.55 × 10^−5^). Miyaki et al. [49] also reported C-344 T SNP of the CYP11B2 gene12 and C1117A SNP to be associated with hypertension in Japanese men. Their study confirmed that the strong association between COMT (rs4680 and rs4633), ATP2B1 (rs17249754) and CYP17A1 (rs11191548) and hypertension [49]. In a study among African Americans, Adeyemo et al. [50], found PMS1, SLC24A4, YWHA7, IPO7, CACANA1H, SLC24A4 and CACNA1H genes to be involved in blood pressure regulation.

GWAS has allowed the discovery of rs9349379 (in PHACTR1), rs1630736 (in EDN1) and rs10305838 (in EDNRA), all of which are linked to the endothelin pathway in hypertension. Polygenic risk scores (PRS), which represents the sum of risk alleles from GWAS has been useful for predicting hypertension. For example, the genetic risk score was used to show the association between blood pressure and coronary heart disease, stroke, and heart failure [51, 52]. Individuals with the highest 2.5% PRS were also shown to have 2.3-fold risk of hypertension in the FinnGenn population based-cohort study. The study further indicated a 10.6 years earlier hypertension amongst individuals with high PRS when compared to those with average PRS [53].

The major bottle neck is associating SNP to causal genes and function. There is only limited evidence to demonstrate the clinical translation of GWAS. Additionally, GWAS information on blood pressure cannot be generalised since it has mainly been applied to Caucasians, with limited studies amongst non-Caucasians. Moreover, interpretation of GWAS can be challenging because non-coding variations may be associated with a phenotype. Lastly, GWAS primarily focuses on common and low-frequency SNPs whereas newer technologies allow the identification of rare variants with large associations with BP [46].

**Fig. 2:**
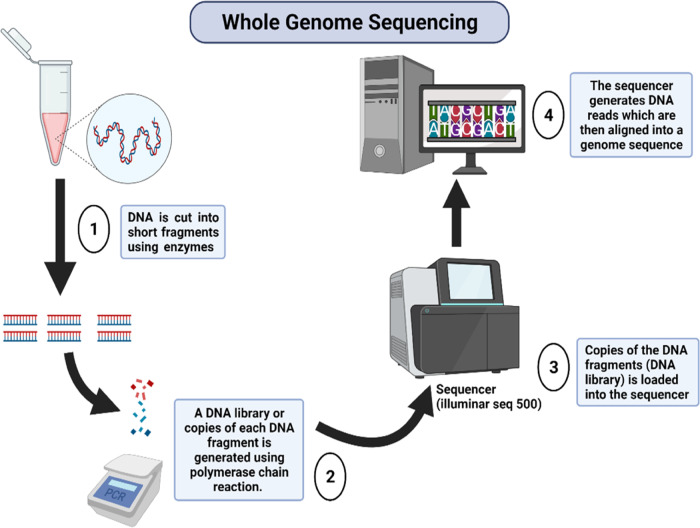
The whole genome sequencing. This procedure involves DNA isolation, library preparation, sequencing, and data analysis.

The advent of technologies has allowed the genetic analysis possible, and disease-causing variants can be detected. Florescent in situ hybridisation (FISH), which involves probes binding to complementary nucleic acid sequence on target cells, enables the detection of cytogenic abnormalities and the expression of pathogens in affected cells [54]. However, because FISH can only detect known imbalances, it is not ideal for screening chromosomal rearrangements [55]. Also, a lack of signal amplification may impact its sensitivity [54]. Today, interest in genetics has heightened, driven by high throughput technologies that have allowed an unprecedented and simultaneous exploration of multiple genes in a single assay. Whole genome sequencing (WGS) **(**Fig. 2**)** and whole exome sequencing (WES) are powerful tools for analysing and providing a high-resolution overview of the entire genome or the exome, respectively [56]. They can reveal large and small variants and provide a comprehensive data information about the genome. Aided by WGS, Wang et al., (2020) reported a 3 rare loss-of-function variants in the PTGIS and showed that this variant was associated with idiopathic pulmonary arterial hypertension [57]. Tran et al. [58], explored the association of common and rare variants in proprotein convertase subtilisin/kexin type 9 (encoded by the PCSK9 gene) and found SNPs (rs12048828: β = 1.8, *p* = 0.05 and rs9730100: β = 1.0, *p* = 0.05) to be associated with DBP. To identify low-frequency and rare variants associated with hypertension, He et al. [59], analysed the coding and rare non-coding variants in the 16p13 region of 30,383 subjects who were part of the Trans-Omics for Precision Medicine WGS project. The study found variants in SLX4 (*p* = 2.19 × 10^−4^) and RBFOX1 (*p* = 0.007) to be associated with BP traits [59]. From the Exome Chip data on 2045 African American participants, Sung et al. [60], highlighted the results of rare and low-frequency single variants and four sets of gene-based analyses. However, both single nucleotide variants (SNVs) and gene level analyses could not reach statistical significance after Bonferroni-correction (*p* < 6.4 × 10 ^−7^ for SNVs; *p* < 2 × 10^−6^ with MAF < 1% and *p* < 3.9 ×  10^−6^ with MAF < 5%). When 135 Exome Chip SNVs were investigated for their associated with cardiometabolic traits, three common variants BRAP, ACAD10, and ALDH2 genes within the 12q24.12 locus were identified to be significantly associated with both SBP and DBP [61].

The WGS is supported by next generation sequencing (NGS). NGS is useful in several ways: 1) Pathological variants in the genome (coding and non-coding) can be identified and sequenced, offering an opportunity for the design of drugs that target such variants [14]. 2) It enables the determination of the causal link between the genotype and the phenotype or the human genome as a whole. 3) The contribution of the environment to disease process can also be determined. Some limitations of NGS include the high cost of equipment and analysing unspecified variants can be complicated [62].

PPPM thrives with a comprehensive knowledge of the genetic information or genetic make-up of individual (genotype) and the corresponding manifestation in disease state (phenotype) or patient symptoms. This is particularly true since there is inter-individual variability in therapeutic response and disposition. When individuals are grouped based on their genetic susceptibility towards a disease, they can be isolated for treatment and tailored prevention strategies can be instituted. Candidate genes can be tested for drug-patient interactions that in turn allow the design of drugs that will be favourable for all disease phenotypes [63].

## Transcriptomics

Transcriptomics involves the study of the complete set of RNA transcripts from the genome of an organism. Gene expressions are tightly regulated and are crucial for cell growth and differentiation [64]. Analysis of transcripts is not new as by the 1970s, molecular biologists could construct RNA libraries and use reverse transcriptase to convert mRNA to complementary DNA (cDNA). By 1980s, individual transcripts were sequenced with Sanger sequencing method [65]. The Sanger method became less popular when other advanced methods such as northern blotting and reverse transcriptase quantitative polymerase chain reaction (RT-qPCR) were developed. Northern blotting technique is inexpensive, does not require a specialised equipment and can adequately determine small sized RNA. However, it is a low throughput technique and has low sensitivity [62]. Comprising steps of DNA denaturation, primer annealing and extension, the RT-qPCR technique make use of primers, DNA polymerase, specific ions, and DNA template to amplify a specific fragment of DNA. There are several advantages of this technique, which include efficiency in detecting and quantifying target DNA, a wide dynamic range for quantification and its application is associated with less cross contamination. However, there is a risk of bias with its use and it cannot distinguish between live and dead cells [66]. Other high-throughput technologies such as the microarray methods and sequence-based approaches are used to simultaneously quantify and study components of transcripts such as small RNAs, non-coding RNAs and mRNAs, along with their splicing sites and how they modified post-translationally. Defined as the process by which variable spliced mRNAs are formed from distinct combinations of splice sites within messenger RNA precursor (pre-mRNA), alternative splicing is one of the post-translational modification mechanisms that increases the complexity of gene expression. This process has been reported to occur due to exonization of transposable elements, constitutively spliced exons, and exon shuffling. Alternative splicing has significant effect on protein function including its localisation, interaction with nucleic acids and other proteins [67]. Splicing mutations account for at least 14% of illness-causing mutations, establishing splicing polymorphisms as disease susceptibility markers. The role of alternative splicing in hypertension has been exemplified in the study by Zhou et al. [68]. Their study reported that calcium influx that is facilitated by activated voltage-gated calcium channel CaV1.2 in vascular smooth muscle cells, has a potential effect on myogenic tone and regulation of blood pressure. According to the study, splicing factor Rbfox2 modulate the function of CaV1.2 channel, and highlighted that in hypertensive arteries, the proportion of CaV1.2 channel with alternative exon 33 decreased by 10.5% whereas alternative exon 9 increased by 10.3% [68].

**Fig. 3:**
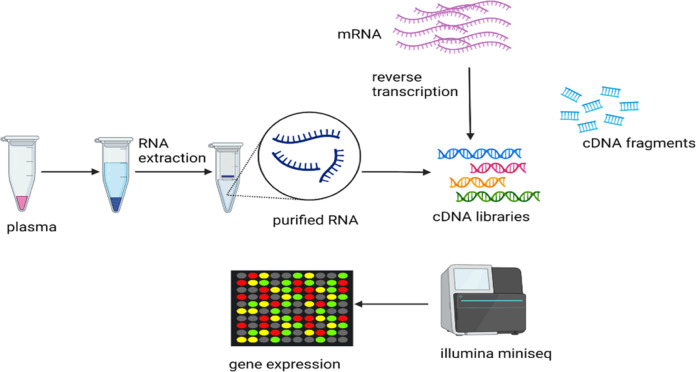
Workflow of transcriptomics. Transcriptomics analysis begins with the extraction of DNA from plasma. Reverse transcriptase is then used to obtain complementary DNA fragments. The DNA library is then loaded into the sequencer and analysed. Gene expression can be visualised in heatmaps.

Currently, RNA sequencing (RNAseq) has been developed which has a leverage over traditional methods and provides more comprehensive information including the connection between exons, the location of transcription boundaries, alternative splicing sites, fusion genes and sequence variations [69]. RNAseq is useful for determining genes that are expressed differentially in distinct populations; how normal and diseased tissues compare, and which genes respond to pharmacological agents. Transcriptomics analysis involves isolation and purification of RNA from samples, followed by conversion from RNA to cDNA with reverse transcriptase. The cDNA is loaded in a sequencer and differential expressions are analysed. Differences in gene expression are usually displayed in heatmaps **(**Fig. 3**)**. The limitation associated with RNAseq is that sequences in spliced isoforms may be too high, and thus complicate analysis [62]. Exploration of transcriptomics profiles has provided insight into the pathogenesis of hypertension [70]. For example, following meta-analysis, Marques et al. [71] found 143 genes with altered expression in the adrenal gland, the kidney, the artery, and heart of hypertensive rats, and these genes are pivotal in fatty acid metabolism, energy transport and oxidation.

To identify transcripts that were associated with blood pressure, Zeller et al. [72], explored transcriptome-wide profiles of 2549 individuals in two separate populations. This was further replicated in 1990 individuals. From the study, CRIP1, MYADM, TIPARP, TSC22D3, CEBPA, F12, LMNA, and TPPP3 transcripts were identified to contribute for up to 13% of BP variability (95% confidence interval: 8.7–16.2) [72]. Based on RNA-seq-based expression profiles from peripheral blood mononuclear cells collected from patients, Romanoski et al. [73], identified 61 genes that correlated with pulmonary arterial hypertension severity. While scanning gene expression profiles of blood pressure and hypertension in the blood of 7017 individuals, 34 genes were differentially expressed with blood pressure. Among the transcripts were FOS, MYADM, PP1R15A, TAGAP, S100A10, and FGBP2, all of which were regulated by rs3184504 in SH2B3. The study further indicated that the genes discovered accounted for 5–9%- of inter-individual variation in blood pressure [74]. In another study that compared the leukocyte RNA transcripts from treated and untreated hypertensive patients, 680 genes were differentially expressed in the latter, but were not expressed in treated patients. The study further identified the differential expression of blood pressure genes including increased levels of ANP-A receptor, angiotensin II type 1 receptor, endothelin-2, and 3 of the serotonin receptors but a decreased expression of endothelin-converting enzyme-1 [75]. Basu et al. [70], explored transcriptomics profiles of 29 tissues from 565 individuals and showed that pan-tissue transcriptional dysregulation underlie hypertension. They applied Least Absolute Shrinkage and Selection Operator to determine to explore tissue specific transcriptome of hypertensive patients and non-hypertensive individuals. After conducting transcriptomic analysis of seven tissues including nerve-tibial, colon-transverse, pancreas, artery-tibial, adipose-subcutaneous, muscle-skeletal, breast-mammary tissue, it was revealed that gene expression profiles in hypertension patients were different from non-hypertensive individuals [70]. Based on a hypertensive-score, participants were grouped into a diffused and localised group. The study found that the diffused group had more hypertensive associated alterations than the localised group [70].

## Glycomics

This is a new frontier in medicine that studies the presence, structure, and role of complex sugar molecules (glycans) in an organism [76, 77]. Specifically, the enzymatic attachment of glycan to polypeptide sequences―glycosylation, has gained much attention in recent years. Glycosylation is a complex but highly regulated post translational modification process, occurring in the endoplasmic reticulum and the Golgi apparatus. However, this process should not be confused with glycation, which involves the chemical addition of complex sugars to proteins. Glycosylation can be O-linked, where monosaccharides are pinned to serine and threonine hydroxyl groups, C-linked where indole ring of tryptophan is complexed with mannose, and N-linked, where glycans are bound to amino group of asparagine residues. Amongst all these glycosylation types, N-glycosylation is the most researched, impacting up to 90% of all glycoproteins [78]. When attached to a protein, the sugars change the protein’s half-life, trafficking, folding and turnover.

**Fig. 4:**
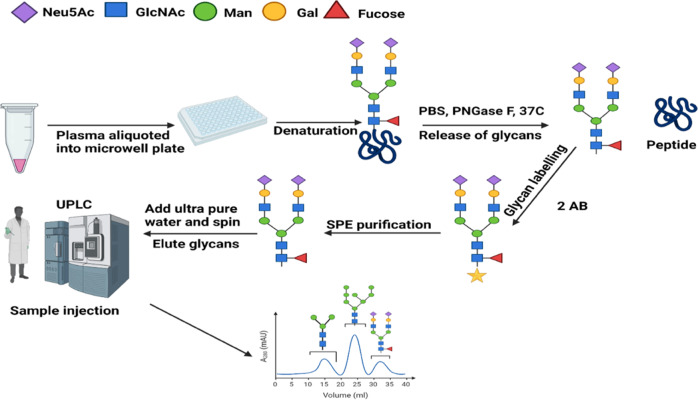
Workflow of N-glycan analysis with UPLC-FLR. Plasma samples are aliquoted into 96 well plates and denatured with sodium dodecyl sulfate (SDS). The plate are sealed and incubated at 65°C for 10 min. IGEPAL CA-630 is added and sample mixed by pipetting up and down. This is then followed by incubation at room temperature. Glycans are freed from their bound glycoproteins by adding peptide N-glycosidase F (PN-Gase F) and incubated at 37°C for 18 hr. Glycans are then fluorescently labelled with 2-aminobenzamide and incubated for 2 hr at 65°C. This is followed by four-step washing procedure with acetonitrile and 2AB glycans are eluted using ultra-pure water. Samples are injected into the UPLC and analysed under the following conditions: solvent A = 100Mm ammonium formate, solvent B = acetonitrile, flow rate 0.1 ml/min, pH = 4.4. Structural assignments and normalisation of glycan peaks are then performed.

The composition of the human N-glycome, evaluated and quantified by analytical technologies, can offer new insights in hypertension pathogenesis. N-glycans are first freed from their bound glycoproteins enzymatically by peptide-N-glycosidase (PNGase) F or N-glycanase, peptide N-endoglycosidase or chemically by β-elimination. Since glycans are non-UV absorbing molecules, they are labelled to enhance detection [17, 76]. Popular labelling tags include 2-aminobenzamide, 2,6-diaminopyridine 2-aminobenzoic acid, amongst others. Following this, structural assignment and annotation are achieved by database searching (Fig. 4). Several analytical approaches are available for quantification and analysis. These include capillary electrophoresis (CE) [79, 80], nuclear magnetic resonance imaging [81], mass spectrometry [82], and ultra-performance liquid chromatography (UPLC) [17]. While MS technologies provide adequate structural elucidation, and facilitate site-specific glycosylation with high sensitivity, they are characterised by sialic acid loss and cannot separate structural isomers. CE is efficient for isomer separation but not ideal for site-specific glycosylation analysis. UPLC has shorter analytical runs, has good resolution and lower solvent consumption. However, they have poor loading capacity and retention due to low surface area. NMR requires minimal sample preparation and efficient for non-selective analysis but intrinsically insensitive [18, 76, 83, 84].

N-glycan profiles can serve as dynamic indicators of the ageing process and are able to discriminate between normal and accelerated ageing by highlighting a discrepancy between a body’s age in years of life and its age in terms of health status. The alteration in their composition in the human glycome reflects a defective cardiometabolic process, akin to many chronic diseases including hypertension [17]. The role of inflammation in the pathophysiology of hypertension has been documented, and this can be explained by glycosylation of immunoglobin G (IgG). IgG is a glycoprotein that links the innate to the adaptive immune system and has been identified to elicit both inflammatory and anti-inflammatory responses. The presence of glycan moieties on the Fc domain of IgG may affect its affinity to Fc receptors (FcRs) and hence, affect the effector functions of IgG. Indeed, complexing the terminal end of IgG Fc *N*-glycan with sialic acid (*N*-acetylneuraminic acid (Neu5Ac) reduces its affinity for FcγRIIIa and results in an anti-inflammatory response by inhibiting antibody dependent cytotoxicity (ADCC). Aberrant Fc N-glycopeptide profiles of plasma IgG subclass has been linked to hypertension. For example, using nano UPLC coupled with MS to quantify plasma samples from Kazakh population, Gao et al. [85], showed that 14 IgG subclass-specific Fc N-glycopeptide and one derived N-glycan trait was associated with SBP and DBP.

The association between hypertension and IgG glycosylation was investigated in a cross-sectional study in different geographical locations including China, Scotland and Croatia. Enabled by hydrophilic interaction chromatography of fluorescently tagged glycans, researchers investigated N-glycans bound to IgG in plasma samples from 4757 people of Chinese Han, Croatian, and Scottish ancestry. The study found five IgG glycosylation traits (G2n, glycan peak (GP) 12, GP14, GP12n, and GP14n)), all being digalactosylated, to be lower in prehypertension [19]. In addition, 17 glycan traits were differentially expressed in normotensive individuals compared to hypertensive patients. Four glycan traits including bisecting GlcNAc, GP4, GP9 and GP21 were found to be associated with hypertension in the TwinsUK, Dalmatians and KORA cohorts [20]. Robajac et al. [86], reported that reduced fucosylation of glycans, along with an increase in paucimannosidic and mannosidic structures were present in human placental membranes, illustrating the potential role glycosylation in preeclampsia. Although there are only limited studies that investigate the role of glycosylation and hypertension, approaches to target key enzymes such as glycosyltransferases/hydrolases, as well as sugar nucleotides and glycoconjugates/glycoforms could enhance our understanding of the pathophysiology of hypertension.

## Proteomics

**Fig. 5:**
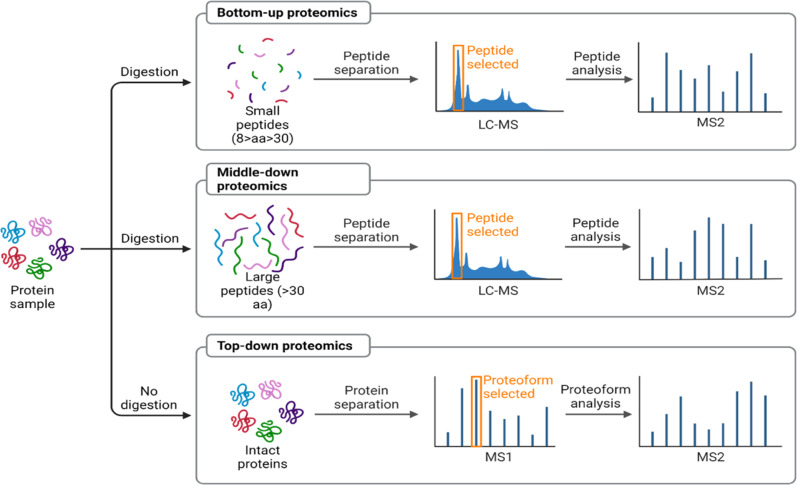
Proteomics analysis. For top-down proteomics where intact proteins are resolved by 2D PAGE and individually characterised later. In bottom up, proteins are digested by proteolytic enzymes and the separated peptides are analysed by MS (Figure modified from Kennani et al. [111**]**).

Proteomics is the study of the set of proteins encoded by the genome, along with protein isoforms, interaction between them, localisation and modification [87, 88]. Proteomics provides the opportunity to understand aberrant protein expression, therapeutic potential of proteins and how new biomarkers for chronic diseases can be developed. The burgeoning interest in proteomics has been provoked by large-scale DNA sequences, and the realisation that focussing on only the genome provides only a partial information about the aetiology of diseases. Proteins are products of gene translation and are involved in the many metabolic and regulatory pathways in the cell. However, since there are an estimated 100,000 protein isoforms from 20,325 genes, proteomics analysis constitutes a formidable challenge thus, requires high throughput and sensitive technologies [89]. The first protein expression profiling was achieved in the mid-70s using two-dimensional polyacrylamide gel electrophoresis (2D PAGE) [90]. In the 2D PAGE, proteins are separated by their net charge and molecular mass in the first and second dimensions, respectively [91]. While 2D can resolve proteins, profiling is slow and laborious, and it lacks sensitivity. This represents the so-called top-down proteomics where intact proteins are characterised. This approach is suitable for determining protein isoforms and for post-translational modifications. However, the protein ionisation and fractionation are major bottlenecks. Nowadays, MS Shotgun (bottom-up) proteomics is possible, and, in this method, proteins undergo proteolytic digestion with trypsin and the separated peptides or fragments are analysed by tandem MS/MS **(**Fig. 5**)**. This approach has leverage over the top-bottom method since peptides can be easily ionised and fractionated. The era of mass spectrometry has revolutionised proteomics research, impacting our understanding of the complexity of many chronic diseases. Peptides in plasma samples can be separated on NanoLC 425 System (SCIEX) and mass spectrophotometric analysis can be done with SWATH acquisition methods. Like other “OMICS”, the amount of data generated from MS based proteomics is enormous, thus requiring advanced statistical methods for analysis.

Proteomics provides a comprehensive understanding of hypertension, allowing a more precise approach for diagnosis and treatment. Proteomics has a leverage over genomics in navigating the complexities that underlie hypertension. First, proteomics allows the quantification of multiple proteins all at once. Secondly, proteomics allows the determination of information that is not possible with genomics alone, or mechanisms of diseases that are not gene dependent. Third, proteomic analysis is an intermediate between gene expression and cellular function [92]. To understand the interaction between salt intake and hypertension, Matafora et al. [93], conducted proteomics analysis on urine samples. In this study, patients were infused with saline and were divided into salt-sensitive and salt resistant. Their study revealed that salt sensitive patients regulated RAAS differently from salt resistant patients, although there was no difference in Na^+^ reabsorption for both groups. Moreover, the authors showed that glutamyl aminopeptidase, plasminogen activator, urokinase, epidermal growth factor and Xaa-Pro aminopeptidase 2 precursor regulate ENaC-dependent sodium reabsorption. Performing proteomics analysis with LC-MS/MS, de la Cuesta et al. [94], identified two proteins kalirin and chromodomain-helicase-DNA-binding protein 7 to be associated with endothelial dysfunction in hypertensive patients with albuminuria. Proteomic profiling of urinary peptides using CE-MS, Kuznetsova et al. [95], identified 85 discriminating biomarkers for left ventricular diastolic dysfunction. To evaluate left ventricular hypertrophy, Jin et al. [96] employed 2D CE-MS to examine protein expression in the left ventricular myocardium of spontaneously hypertensive and control Wistar Kyoto rats. In spontaneously hypertensive rats, they detected 13 proteins that were expressed differentially before the onset of hypertension. Along with proteins linked to mitochondrial oxidant phosphorylation, oxidative stress, and cellular energy metabolism, two key glycolysis enzymes, A-enolase, and lactate dehydrogenase B, were discovered. Sandrine et al. [97], applied surface-enhanced laser desorption/ionization time-of-flight (SELDI-TOF) mass spectrometry to look for markers of hypertension-related morbidity. The study found ubiquitin, smooth muscle (SM) 22, thymosin 4, and the C-terminal portion of filamin A as the four mass-to-charge ratio (m/z) peaks that were differently released in Fischer rats who were given a low dose of the hypertensive drug N(Q)-nitro-L-arginine methyl ester.

## Lipidomics

**Fig. 6:**
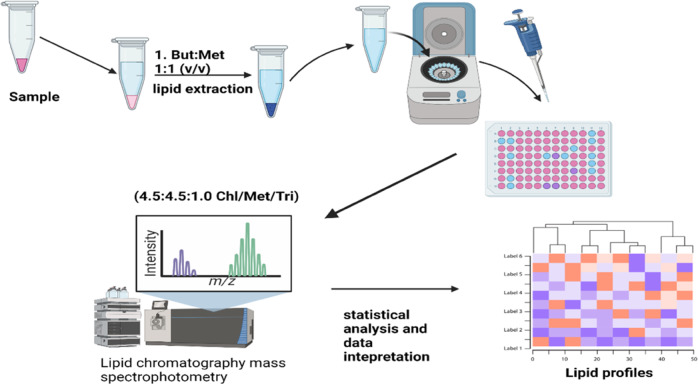
Lipidomic analysis. Lipids are extracted using based on liquid-liquid extraction using various organic solvents. When loaded unto HPLC, analytes can be separated based on their interaction with the stationary and mobile phases. With an appropriate MS analyser, precursor selection can be achieved and fragmented in MS/MS.

The search for novel biomarkers has focussed on genomics, but lipidomics now offer fresh vistas for innovation in hypertension biomarkers. Lipidomics encompasses the large-scale study of the structure and different molecular species of lipids, their cellular and tissue distribution, and their functions in metabolic pathways. Amphipathic lipids form the bilayer of cell membranes, ensuring membrane fluidity and regulating membrane proteins. Hydrophobic lipids on the other hand, are involved in energy metabolism and the regulation of fatty acid storage and release. There are different classes of lipids including glycerols, fatty acyls, sphingolipids, glycerolipids, sterol and prenol lipids, polyketides and glycerophospholipids, saccharolipids [98–100]. Lipids are diverse, and currently there more than 180,000 lipid molecular species. Due to its diversity, identification and analysis require advanced technologies. Lipidomics analysis can be achieved by chromatographic based methods such as reverse phase liquid chromatography. Plasma lipids are extracted based on liquid-liquid extraction using solvents such as butanol and methanol **(**Fig. 6). This is followed by sonicating and centrifugation to precipitate proteins. The non-polar stationary phases comprise silica microparticles with hydrophobic chains whereas the polar mobile phase can be water, acetonitrile, methanol, and isopropanol. When samples are loaded unto the column, lipids species can interact with the hydrocarbons in the stationary phase. As the organic mobile phase is altered, analytes are separated and released based on their affinity or extent of interaction between stationary and the mobile phases [18, 76]. Lately, mass spectrometry (MS) using electrospray ionisation (ESI) and MS-based shotgun lipidomics are applied for large-scale or population level lipidomic profiling. With this technique, multiple complementary scans can be obtained with high accuracy and resolution, providing a holistic exploration of all lipid species. Moreover, high resolution mass analyser such as quadrupole orbitrap, Fourier transform ion cyclotron resonance and quadruple time of flight can be leveraged for precursor selection for MSMS fragmentation and analysis in high resolution [101, 102]. Because MS cannot resolve isomeric species of lipids and has a limited dynamic range, it is useful to couple to LC [102]. Thus, some laboratories use LC–MS based data-dependent acquisition (DDA) and gas chromatography–mass spectrometry (GC–MS) methods for lipidomic analysis. Identification of lipids is based on a software such as LipidXplorer [103]. It is now evident that lipids are not just important for building cell membranes, but they are involved in cell-cell interaction and signalling. Thus, defects in the structure of lipids are linked to various diseases. Until recently, the study of lipids has been restricted to total cholesterol, triglycerides, low density lipoprotein cholesterol and high-density lipoproteins. While these have been the biomarkers for disease states including hypertension, they only provide a partial information. Lipidomic profiling provides a better way of distinguishing lipid levels in health and diseased individuals. In the San Antonio Family Heart Study, Khulkani et al. [104], scanned lipidomic profiles of hypertensive patients with HPLC-MS. Up to 319 lipid species were investigated in 1192 people from 42 big and extended Mexican American families. In another study, plasma ceramides C16:0, C22:0, C24:0, and C24:1 were found to be higher in spontaneously hypertensive rats when compared with normotensive Wistar-Kyoto rats [105]. Ceramides can potentially inhibit endothelial nitric oxide synthase, resulting in endothelial dysfunction in hypertension [106]. Using LC-MS, Egan et al. [21], identified diglycerols (DGs), especially, DG 16:0/22:5 and DG 16:0/22:6 lipid species, to be associated with SBP, DBP and mean arterial blood pressure. Bivariate trait analysis further revealed that these lipid species correlated genetically with the liability of hypertension. A top-down shotgun profiling on a LTQ Orbitrap hybrid MS was used to profile plasma lipidome of hypertensive patients and normal individuals [107]. It was shown that ether phosphatidylcholines and ether phosphatidylethanolamines, having arachidonic (20:4) and docosapentaenoic fatty acid moieties, as well as free cholesterol were lower in the plasma of hypertensive patients [107]. Also, hypertensive patients were shown to have higher levels of triglycerides while another study reported hypertensive rats had increased levels of ceramides than controls [105]. Liu et al. [108], applied electrospray ionisation tandem MS to quantify plasma phospholipids in five European cohorts. They found six phosphatidylethanolamines (PE 38:3, PE 38:4, PE 38:6, PE 40:4, PE 40:5 and PE 40:6) and two phosphatidylcholines (PC 32:1 and PC 40:5) that were associated with hypertension. Performing plasma lipidomics and multivariate analysis, Hu et al. [109], showed that the metabolism of lipids in hypertensive patients was different from healthy individuals. Phosphatidyl choline and triglycerides were elevated in hypertensive patients.

## Conclusion

Hypertension, characterised by constant rise in systemic blood pressure, is a function of cardiac output and peripheral vascular resistance. Different physiological systems including renal, endocrine, and neuronal affect the regulation of blood pressure. These must be tightly regulated to achieve a normal blood pressure status. Hypertension’s multifactorial and asymptomatic nature makes it difficult to identify those who will acquire the condition. Subsequently, hypertensive patients have struggled to achieve the recommended blood pressure targets, making them susceptible to other comorbidities. The advent of high-throughput analytical multiomics technologies has provided a means to identify robust biomarkers that would bring precision to medicine. In this review, aberrant genes, proteins, lipids, sugars that underlie hypertension pathophysiology have been highlighted. While the genome largely stays the same from conception to death, the transcriptome, lipidome, proteome and glycome may be modified from altered cellular environment and disease status. Therefore, they serve as a link between our cells’ genetic makeup and their cellular environment, which is heavily influenced by our daily behaviours and routines [27]. Yet, the translation into clinical practise has been slowed due to complexity in interpreting data and the cost associated with OMICS studies. In addition, most studies cited in literature are cross-sectional, providing limited information about cause-effect relationships. Thus, multi-omics integrative analyses involving continuous variations in blood pressure values are required. Nonetheless, multiomics offers a multidimensional way to explore the interplay between internal and external risk variables that underlie hypertension pathogenesis. OMICS technologies have potential positive effects on future clinical practise, including for discovering pathogenic factors, describing the molecular makeup of hypertension and for monitoring response to treatment [110].
